# Supplementary material for: Imaging vesicle formation dynamics supports the flexible model of clathrin-mediated endocytosis
Source: Nat Commun. 2022 Apr 1;13:1732. doi: 10.1038/s41467-022-29317-1 (PMC8976038; doi:10.1038/s41467-022-29317-1)

Supplementary Information

Imaging vesicle formation dynamics supports the flexible model of clathrin-mediated endocytosis.

Tomasz J. Nawara[1], Yancey D. Williams II[1], Tejeshwar C. Rao[1], Yuesong Hu[2], Elizabeth Sztul[1], Khalid Salaita[2] and Alexa L. Mattheyses[1*]

[1]Department of Cell, Developmental, and Integrative Biology, University of Alabama at Birmingham, Birmingham, AL, USA.

[2]Department of Chemistry, Emory University, Atlanta, GA, USA.

[*]Address correspondence to mattheyses@uab.edu

Contains:

Supplementary Figures 1-10

Supplementary Tables 1-8

Raw western blots

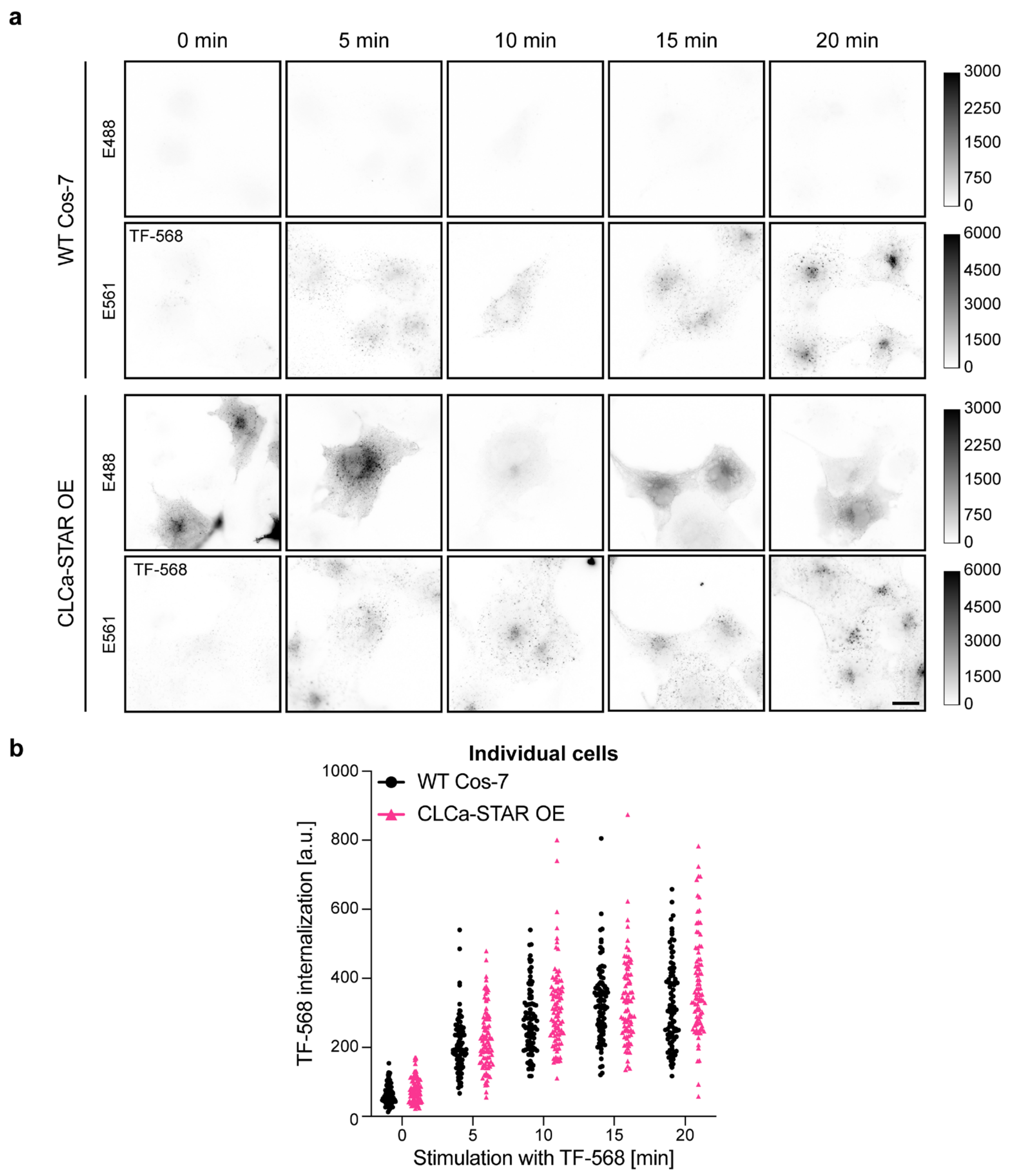

Supplementary Fig. 1: CLCa-STAR does not disrupt overall CME dynamics and transferrin uptake. a Representative Cos-7 cells from each time point of the transferrin-568 (TF-568) uptake assay, E488 – CLCa-STAR, E561 – TF-568. Only the EGFP channel is visualized for both wild-type (WT) and CLCa-STAR overexpressing (OE) cells. **b** Scatter plot of mean TF-568 intensity per cell at 0, 5, 10, 15, and 20 min for WT Cos-7 (n = 93, 93, 93, 93, 93 cells) and for CLCa-STAR OE (n = 89, 91, 93, 86, 92 cells). Data **a** and **b** is from three independent repeats).

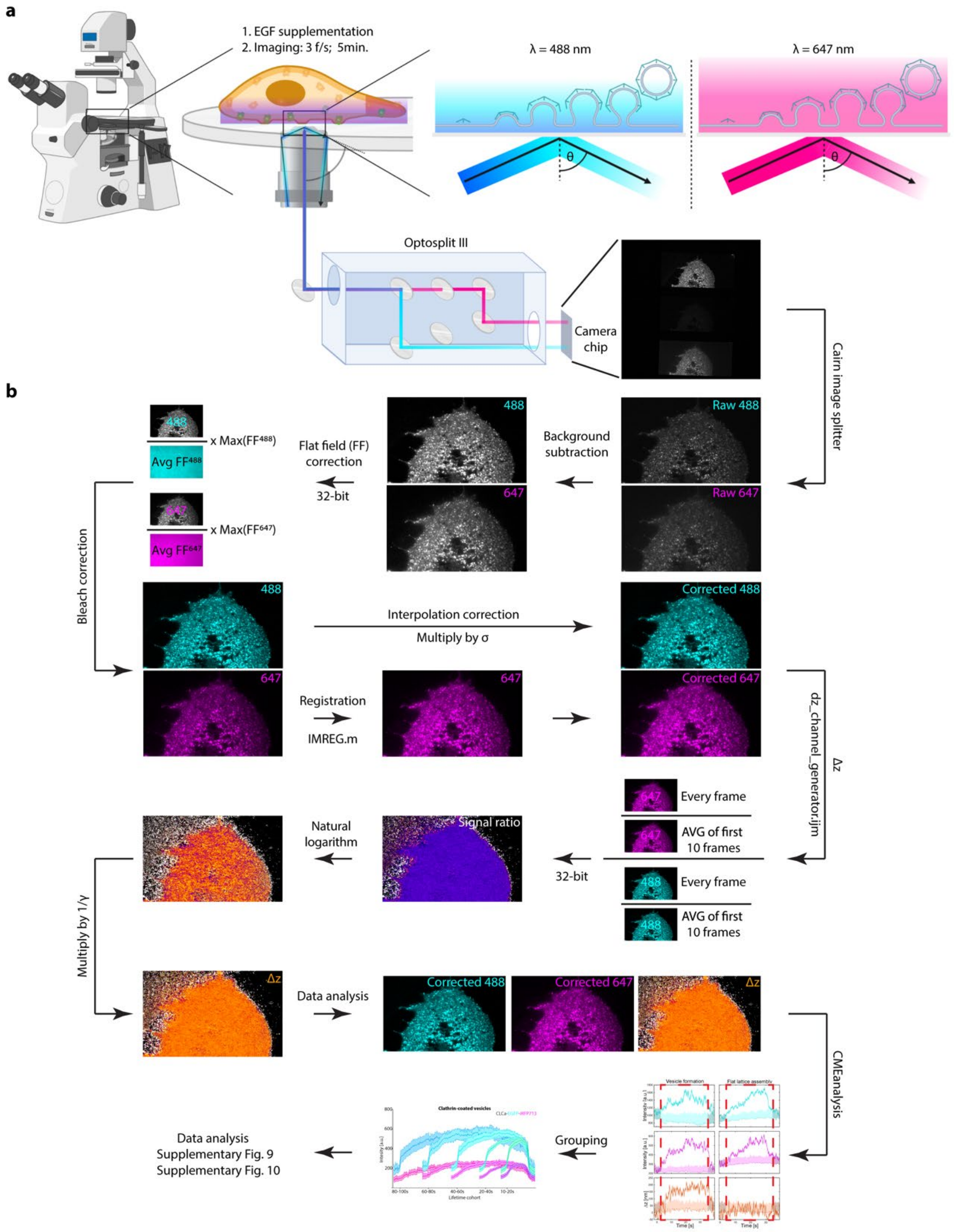

Supplementary Fig. 2: Experimental design, data acquisition, processing, and analysis. a Experimental set up with simultaneous 488 and 647 TIRF imaging (Created with BioRender.com). **b** STAR data processing and analysis pipeline.

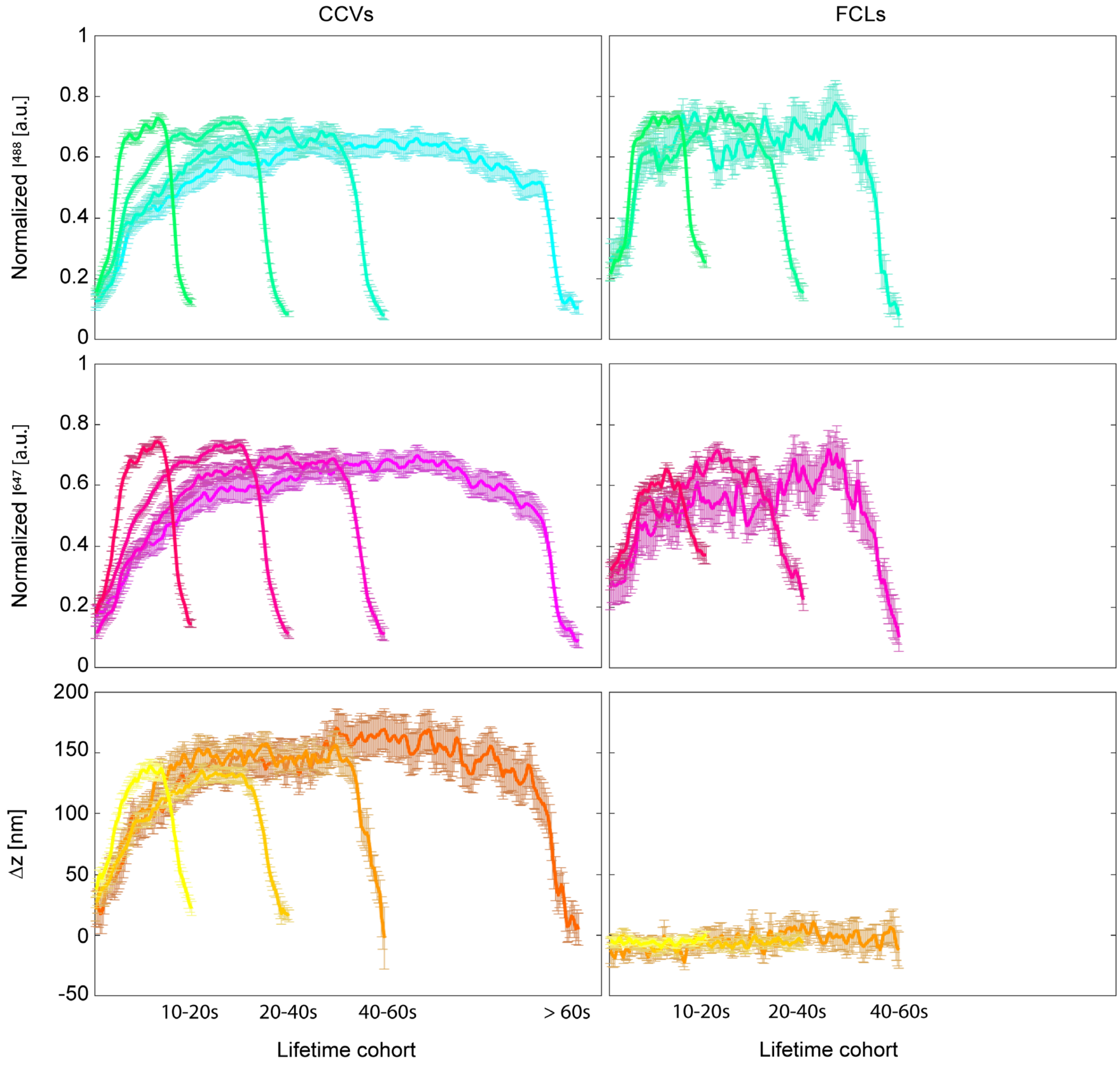

Supplementary Fig. 3: Representative intensity and Δz traces for CMEanalysis identified CCSs. Intensity was normalized for CLCa-STAR in both colors. Means ± SEMs for presented cohorts (CCVs: 10-20s – n = 147, 20-40s – n = 124, 40-60s – n = 40, >60s – n = 28; FCLs: 10-20s – n = 102, 20-40s – n = 34, 40-60s – n = 8, the cohort >60s was not displayed as too few tracks were present).

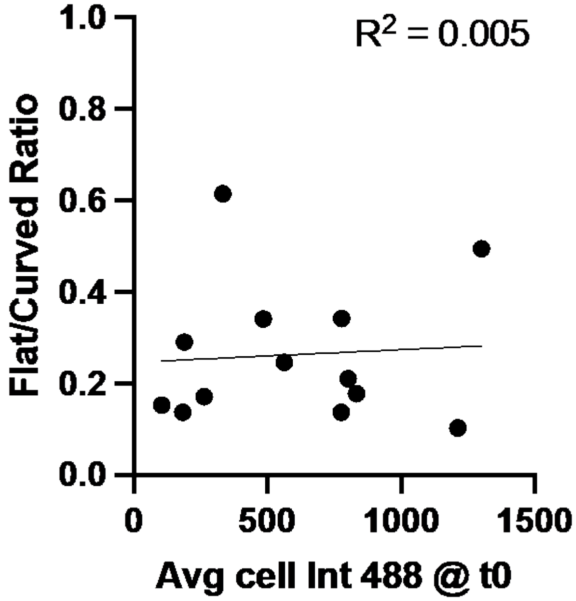

Supplementary Fig. 4: Flat to Curved CCSs ratio is not alter by protein expression level in healthy cells. The flat to curved CCSs ratio was calculated for cells from Figure 2 and was correlated with the overall EGFP intensity of the cell body at t_0. No correlation was found by a simple linear regression (R^2 = 0.005).

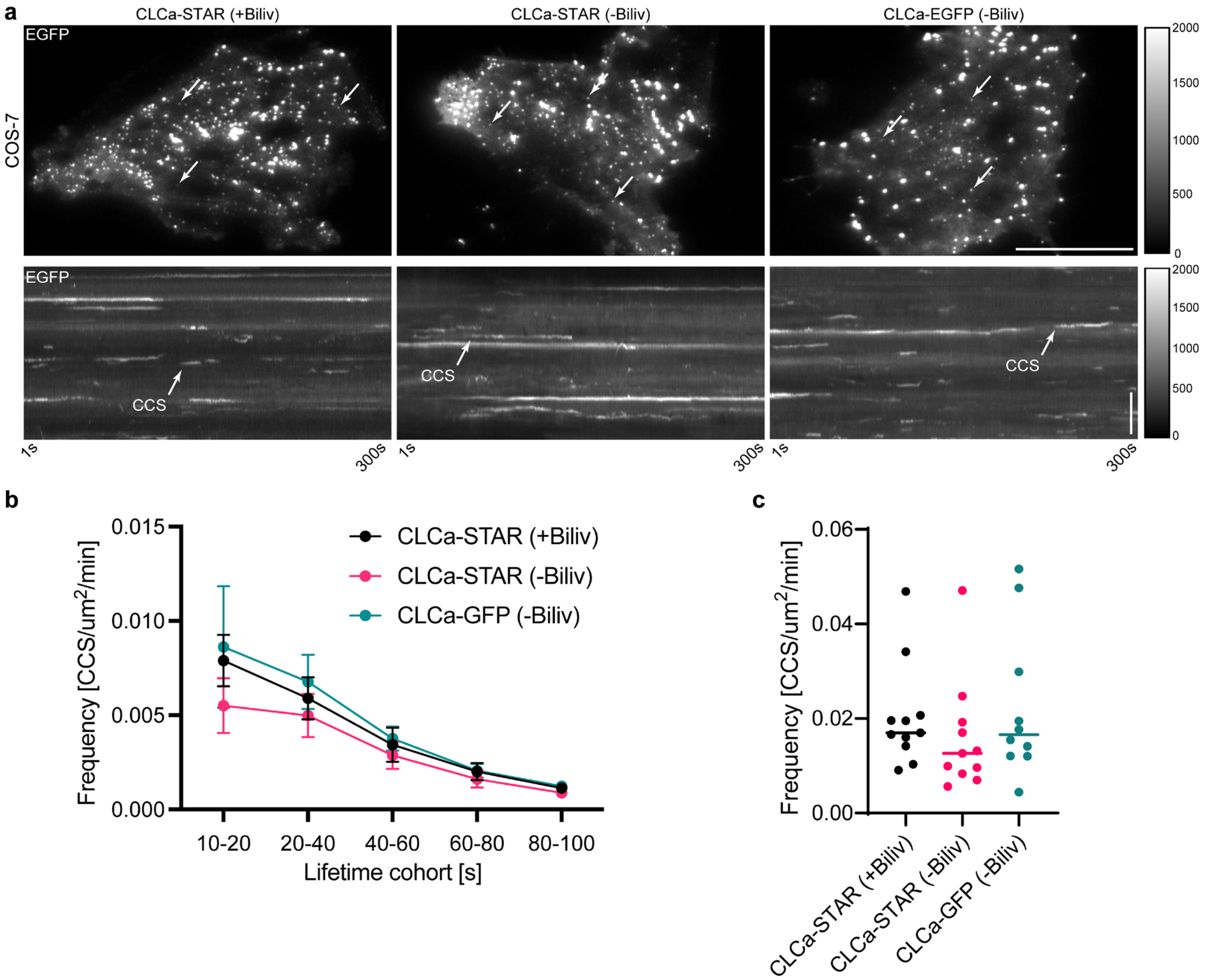

Supplementary Fig. 5: CLCa-STAR, with or without the addition of iRFP713 cofactor biliverdin, behaves similarly to CLCa-EGFP. a Cos-7 cell transfected with CLCa-STAR with biliverdin during starvation - CLCa-STAR (+Biliv), Cos-7 cell transfected with CLCa-STAR without biliverdin during starvation - CLCa-STAR (-Biliv), and Cos-7 cell transfected with CLCa-EGFP without biliverdin during starvation - CLCa-EGFP (-Biliv), imaged using TIRF 488, white arrows point towards example clathrin accumulations, scale bar = 20 µm. Kymographs of clathrin accumulation (gray), white arrows point towards example clathrin accumulations, scale bar = 5 µm. **b** Histogram of lifetime distribution of CCSs per um^2, per minute (mean ± SEM). **c** Cumulative frequency of CCSs per um^2, per minute for each cell. Line – median, data was not normally distributed (one-sided Shapiro-Wilk test, $p < 0.05$ for all three groups), medians were not significantly different as tested by Kruskal-Wallis test, approximate $p > 0.05$; [median, n cells , n events] CLCa-STAR (+Biliv) = [0.01697, 11, 1847]; CLCa-STAR (-Biliv) = [0.01261, 11, 1383]; CLCa-EGFP (-Biliv) = [0.01657, 10, 1805] (Data in **b** and **c** is from two independent repeats).

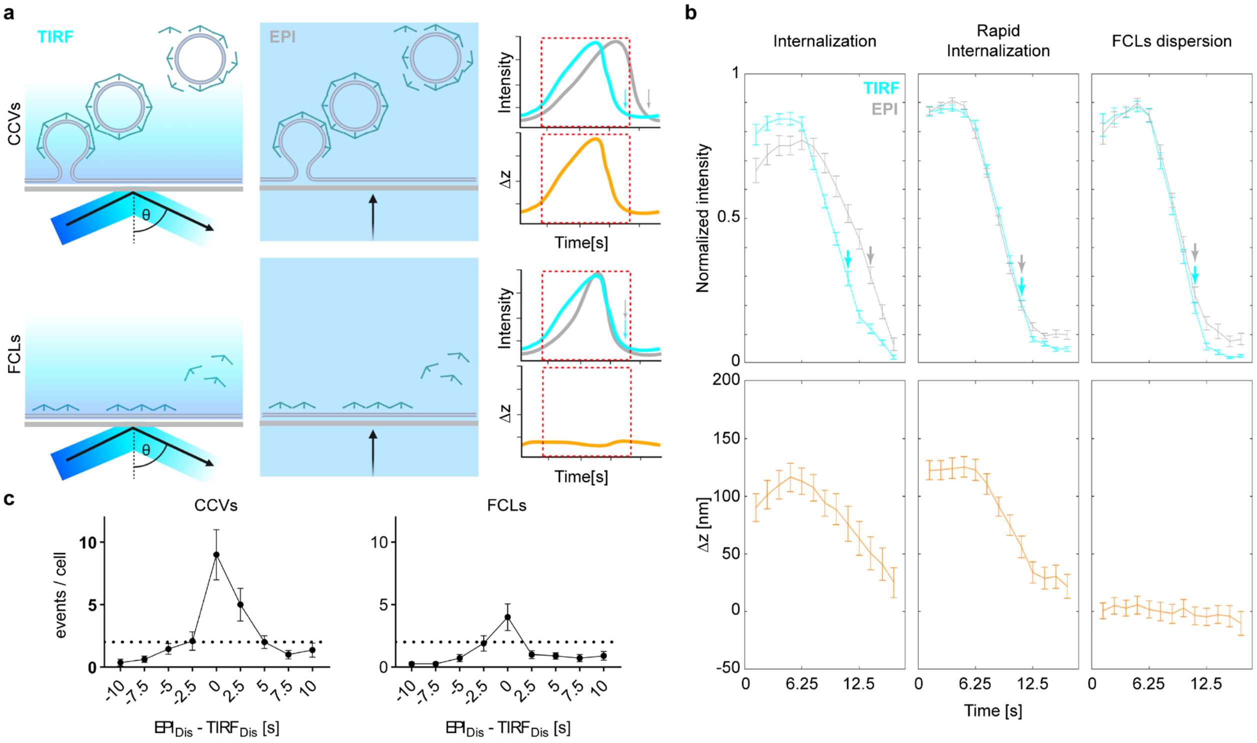

Supplementary Fig. 6: EPI/STAR analysis of CCS dynamics. a Principle of EPI/STAR analysis. As CCVs are internalized, they will disappear from the TIRF excitation field while remaining in EPI and positive Δz changes should be observed. In contrast FCLs, which do not result in endocytosis, should disappear simultaneously EPI and TIRF and no Δz changes should be observed (Created with BioRender.com). **b** Grouping of the EPI/STAR data based on the different signal disappearance dynamics from EPI and TIRF as well as the Δz for those puncta. Intensities are normalized. Means ± SEMs (Representative traces from 2 replicates and 11 cells total: Internalization – n = 62, Rapid internalization – n = 132, FCLs dispersion – n = 44). **c** Plot of the difference distribution between EPI disappearance (EPI_{Dis}) and TIRF disappearance ($TIRF_{Dis}$) of all analyzed puncta separated on whether Δz changes were or were not induced. Data are presented as mean values ± SEM (CCVs – n = 252 events, FCLs – n = 118 events, data from 2 replicates and 11 cells total, cohorts with mean of 2 or less events were discarded from quantifications, the signal disappearance was identified as a first frame when signal reached below background for five consecutive frames).

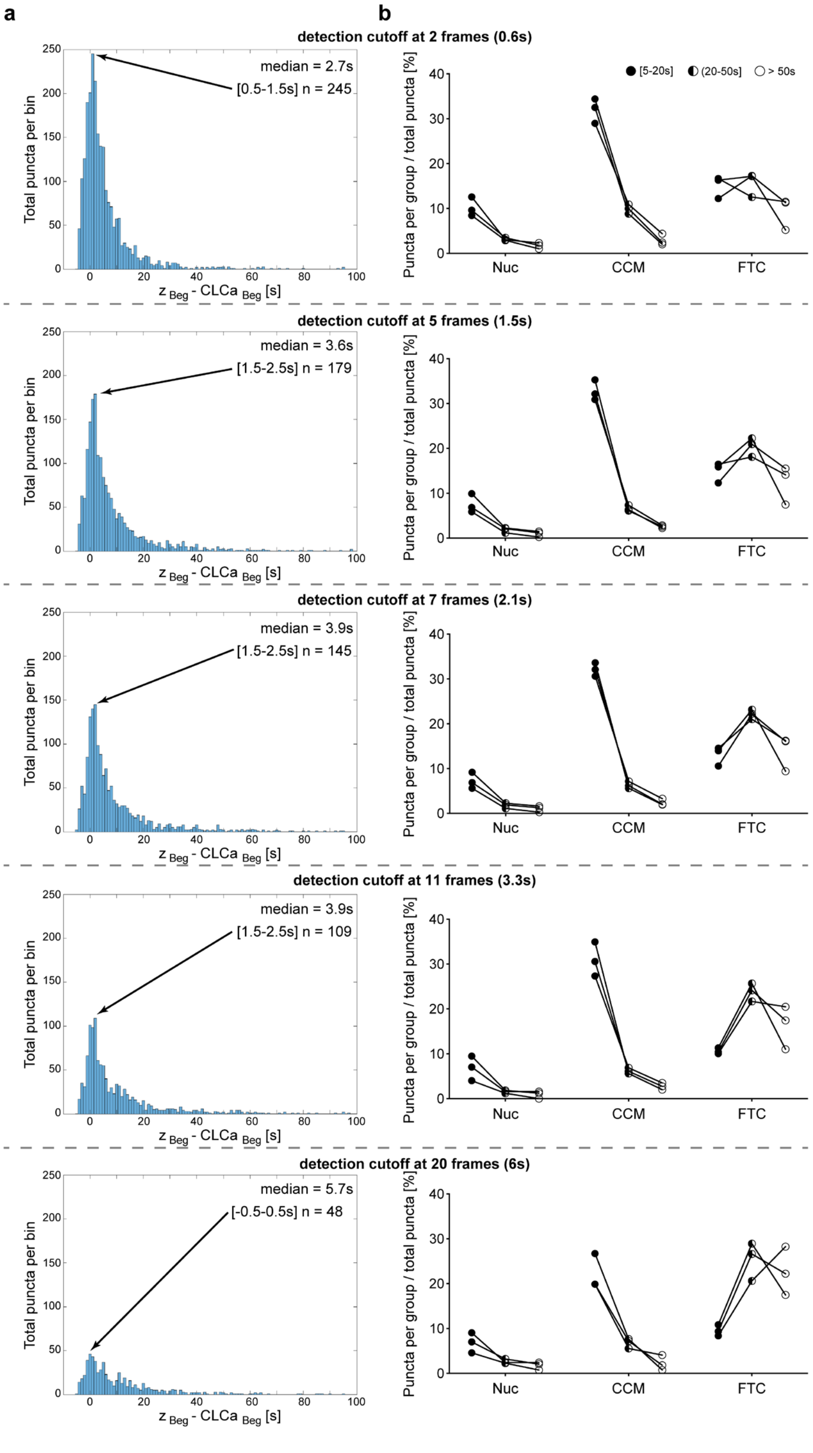

Supplementary Fig. 7: Signal detection cut-off does not explain the variation in curvature formation. a Data from Figure 3 was reanalyzed using a range of detection cut-offs from 2 to 20 frames as indicated. The distribution of Δz_{Beg} - $CLCa_{Beg}$ [s] are shown as histograms for each cutoff. **b** Summary of the distribution of events across the three membrane bending models and lifetime cohorts. Statistical analysis is summarized in Supplementary Table 3.

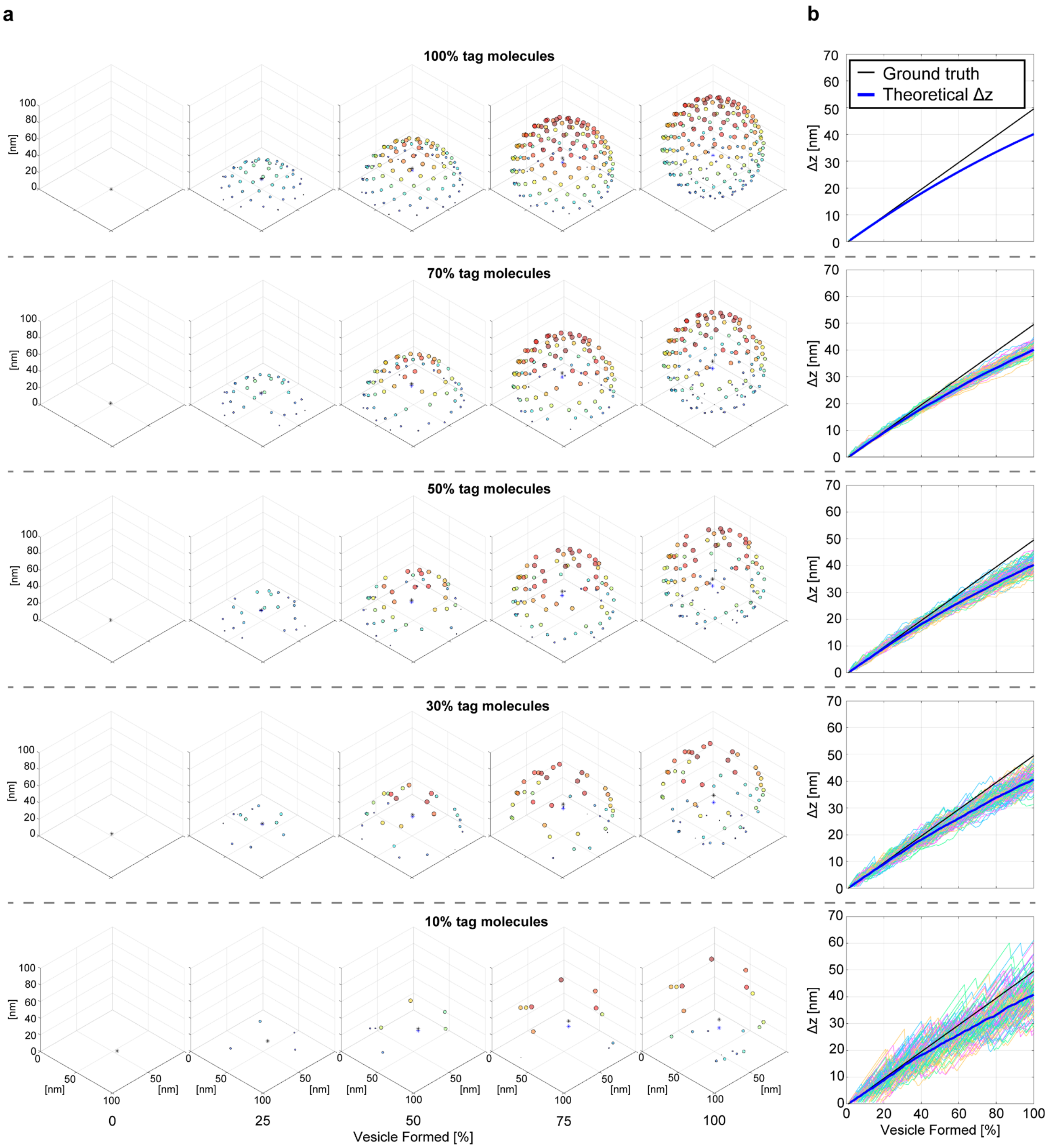

Supplementary Fig. 8: Monte Carlo simulation of STAR measurements, with a variable amount of CLCa-STAR proteins present on the vesicle. a Simulation of vesicle formation with varying percentages of CLCa-STAR tagged proteins distributed randomly on the vesicle; percentage tagged indicated above the plots. **b** Monte Carlo simulation of 100 CCVs (colored lines) and their theoretical Δz measured by STAR microscopy, the thick blue line indicates mean for the simulation and black line indicates the theoretical center of mass (CM) for forming CCVs.

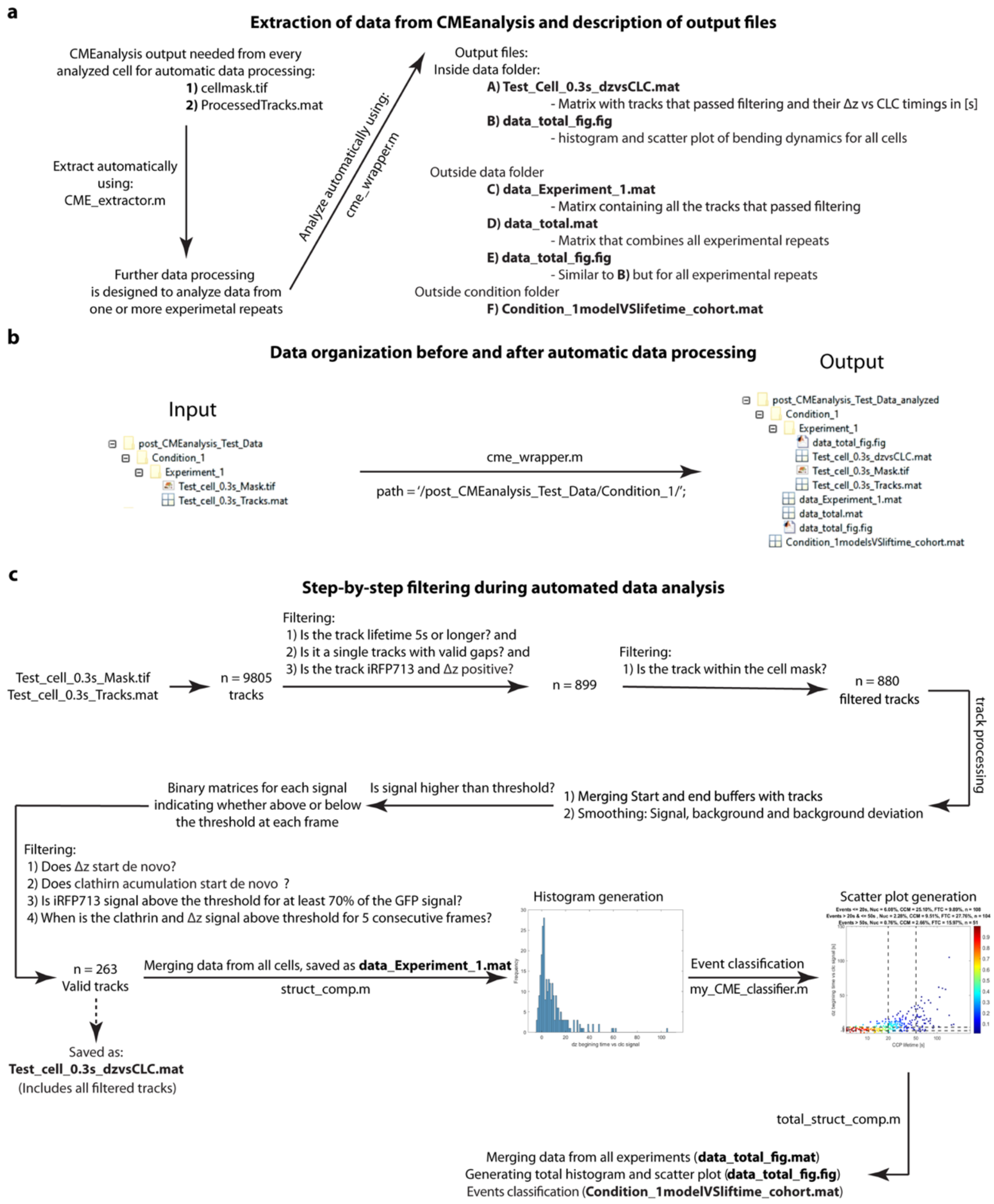

Supplementary Fig. 9: Automated analysis of the initiation of curvature formation. **a** CMEanalysis outputs needed for automated data analysis, and post processing files description. **b** Data organization pre and post automated data processing using the given executable code. **c** Step-by-step explanation of data filtering and visualization.

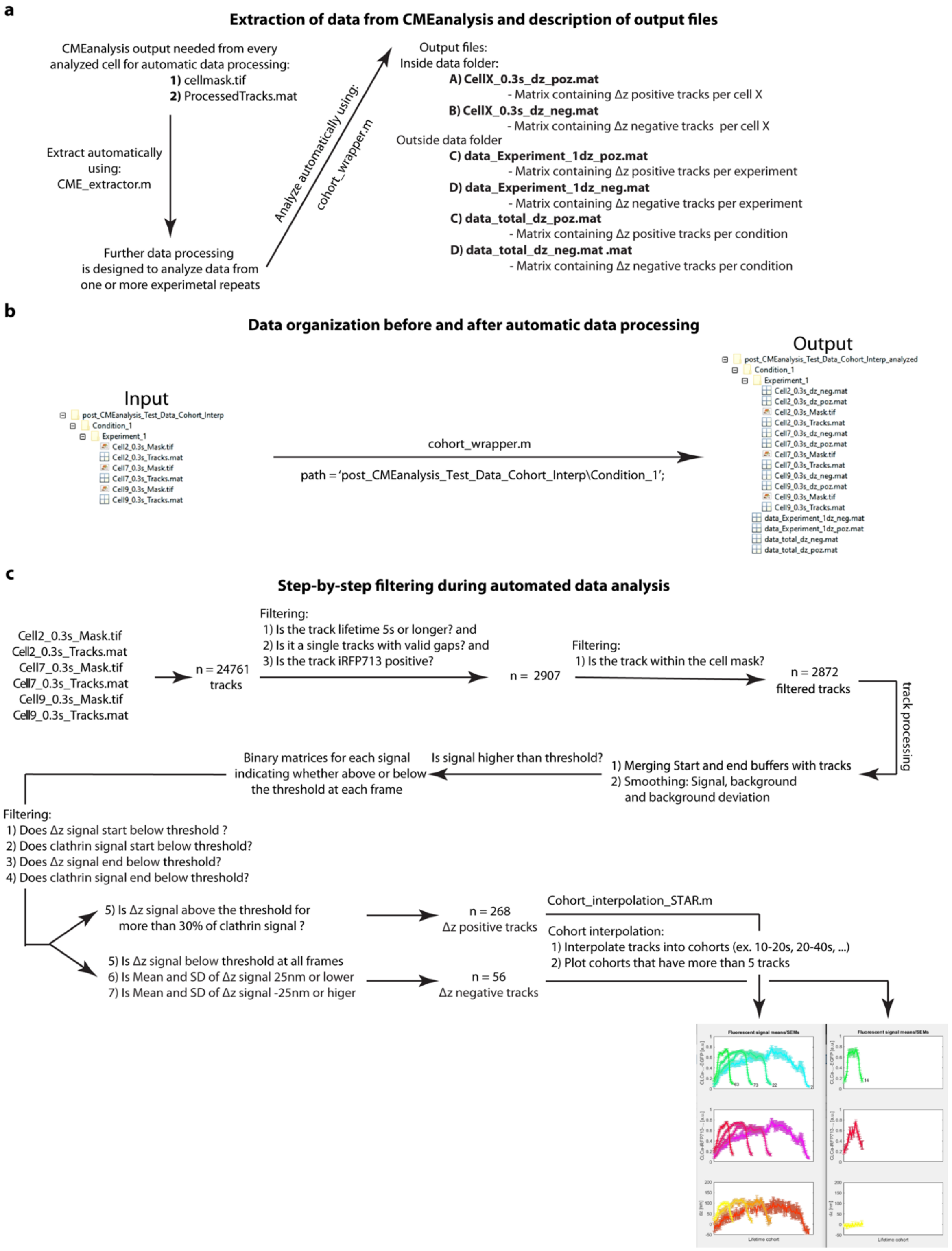

Supplementary Fig. 10: Automated flat and curved events sorting. a CMEanalysis outputs needed for automated data analysis, and post processing file descriptions. **b** Data

organization pre and post automated data processing using the given executable code.
c Step-by-step explanation of data filtering and visualization.

Supplementary Table 1: Šídák's multiple comparisons statistics results for Fig. 1f, post two-way ANOVA. P values were adjusted for multiple comparison using statistical hypothesis testing.

Šídák's multiple comparisons test	Mean Diff.	95.00% CI of diff.	Summary	Adjusted P Value
**WT Cos-7**				
0 vs. 5	-143.9	-433.5 to 145.8	ns	0.1851
0 vs. 10	-214.7	-415.8 to -13.68	*	0.044
0 vs. 15	-268.3	-503.4 to -33.22	*	0.0387
0 vs. 20	-264.5	-594.1 to 65.12	ns	0.0764
5 vs. 10	-70.84	-251.7 to 110.0	ns	0.2776
5 vs. 15	-124.4	-199.8 to -49.05	*	0.0187
5 vs. 20	-120.6	-454.1 to 212.8	ns	0.3156
10 vs. 15	-53.59	-159.6 to 52.38	ns	0.1793
10 vs. 20	-49.78	-224.8 to 125.2	ns	0.4509
15 vs. 20	3.806	-263.4 to 271.0	ns	>0.9999
**CLCa-STAR OE**				
0 vs. 5	-144.7	-412.9 to 123.5	ns	0.1597
0 vs. 10	-233.3	-434.0 to -32.64	*	0.0373
0 vs. 15	-255.6	-442.4 to -68.82	*	0.0271
0 vs. 20	-296.5	-670.2 to 77.12	ns	0.078
5 vs. 10	-88.61	-200.4 to 23.17	ns	0.0782
5 vs. 15	-110.9	-303.9 to 82.12	ns	0.1426
5 vs. 20	-151.8	-263.7 to -39.96	*	0.0275
10 vs. 15	-22.28	-239.7 to 195.1	ns	0.9673
10 vs. 20	-63.22	-251.1 to 124.6	ns	0.3532
15 vs. 20	-40.94	-341.1 to 259.2	ns	0.8897

Supplementary Table 2: Tukey's multiple comparisons statistics results for Fig. 3e, post two-way ANOVA. P values were adjusted for multiple comparison using statistical hypothesis testing.

Tukey's multiple comparisons test	Mean Diff.	95.00% CI of diff.	Summary	Adjusted P Value
Nuc: [5-20s] vs. Nuc:(20-50s]	5.701	-0.2370 to 11.64	ns	0.0655
Nuc: [5-20s] vs. Nuc:>50s	6.58	0.6415 to 12.52	*	0.0235
Nuc: [5-20s] vs. CCM: [5-20s]	-25.23	-31.16 to -19.29	****	<0.0001
Nuc: [5-20s] vs. CCM:(20-50s]	0.9476	-4.990 to 6.886	ns	0.9996
Nuc: [5-20s] vs. CCM:>50s	4.994	-0.9443 to 10.93	ns	0.1415
Nuc: [5-20s] vs. FTC: [5-20s]	-7.338	-13.28 to -1.400	**	0.0094
Nuc: [5-20s] vs. FTC:(20-50s]	-12.88	-18.82 to -6.947	****	<0.0001
Nuc: [5-20s] vs. FTC:>50s	-4.815	-10.75 to 1.123	ns	0.1699
Nuc:(20-50s] vs. Nuc:>50s	0.8785	-5.060 to 6.817	ns	0.9998
Nuc:(20-50s] vs. CCM: [5-20s]	-30.93	-36.87 to -24.99	****	<0.0001
Nuc:(20-50s] vs. CCM:(20-50s]	-4.753	-10.69 to 1.185	ns	0.1806
Nuc:(20-50s] vs. CCM:>50s	-0.7073	-6.645 to 5.231	ns	>0.9999
Nuc:(20-50s] vs. FTC: [5-20s]	-13.04	-18.98 to -7.101	****	<0.0001
Nuc:(20-50s] vs. FTC:(20-50s]	-18.59	-24.52 to -12.65	****	<0.0001
Nuc:(20-50s] vs. FTC:>50s	-10.52	-16.45 to -4.578	***	0.0002
Nuc:>50s vs. CCM: [5-20s]	-31.81	-37.74 to -25.87	****	<0.0001
Nuc:>50s vs. CCM:(20-50s]	-5.632	-11.57 to 0.3061	ns	0.0708
Nuc:>50s vs. CCM:>50s	-1.586	-7.524 to 4.352	ns	0.9874

Nuc:>50s vs. FTC: [5-20s]	-13.92	-19.86 to -7.979	****	<0.0001
Nuc:>50s vs. FTC:(20-50s]	-19.46	-25.40 to -13.53	****	<0.0001
Nuc:>50s vs. FTC:>50s	-11.39	-17.33 to -5.456	****	<0.0001
CCM: [5-20s] vs. CCM:(20-50s]	26.17	20.24 to 32.11	****	<0.0001
CCM: [5-20s] vs. CCM:>50s	30.22	24.28 to 36.16	****	<0.0001
CCM: [5-20s] vs. FTC: [5-20s]	17.89	11.95 to 23.83	****	<0.0001
CCM: [5-20s] vs. FTC:(20-50s]	12.34	6.403 to 18.28	****	<0.0001
CCM: [5-20s] vs. FTC:>50s	20.41	14.47 to 26.35	****	<0.0001
CCM:(20-50s] vs. CCM:>50s	4.046	-1.892 to 9.984	ns	0.3461
CCM:(20-50s] vs. FTC: [5-20s]	-8.285	-14.22 to -2.347	**	0.003
CCM:(20-50s] vs. FTC:(20-50s]	-13.83	-19.77 to -7.894	****	<0.0001
CCM:(20-50s] vs. FTC:>50s	-5.762	-11.70 to 0.1757	ns	0.0611
CCM:>50s vs. FTC: [5-20s]	-12.33	-18.27 to -6.393	****	<0.0001
CCM:>50s vs. FTC:(20-50s]	-17.88	-23.82 to -11.94	****	<0.0001
CCM:>50s vs. FTC:>50s	-9.809	-15.75 to -3.871	***	0.0005
FTC: [5-20s] vs. FTC:(20-50s]	-5.547	-11.49 to 0.3910	ns	0.0779
FTC: [5-20s] vs. FTC:>50s	2.523	-3.415 to 8.461	ns	0.8468
FTC:(20-50s] vs. FTC:>50s	8.07	2.132 to 14.01	**	0.0038

Supplementary Table 3: Signal detection cut-off manipulations and its influence over events classification reported as difference to cut-off used in main text (Fig. 3e)

Mean % tracks	[5-20s]			(20-50s]			>50s			Median for all events	Mode for all events
# of frames to count signal as positive	Nuc	CCM	FTC	Nuc	CCM	FTC	Nuc	CCM	FTC		
2 (0.6s)	10.21 (+2.66)	31.98 (-0.8)	15.08 (+0.19)	3.15 (+1.30)	9.91 (+3.30)	15.68 (-4.76)	1.71 (+0.74)	2.93 (+0.38)	9.35 (-3.01)	2.7s (-0.9s)	[0.5-1.5s] (-1s)
**5 (1.5s)**	**7.55**	**32.78**	**14.89**	**1.85**	**6.60**	**20.44**	**0.97**	**2.56**	**12.37**	**3.6s**	**[1.5-2.5s]**
7 (2.1s)	7.21 (-0.34)	32.12 (-0.66)	13.04 (-1.84)	1.78 (-0.07)	6.31 (-0.29)	22.14 (+1.71)	1.04 (+0.07)	2.44 (-0.12)	13.91 (+1.54)	3.9s (+0.3s)	[1.5-2.5s] (ND)
11 (3.3s)	6.85 (-0.70)	30.95 (-1.82)	10.59 (-4.30)	1.54 (-0.31)	6.21 (-0.40)	23.85 (+3.41)	0.94 (-0.03)	2.76 (+0.21)	16.32 (+3.95)	3.9s (+0.3s)	[1.5-2.5s] (ND)
20 (6s)	6.88 (-0.67)	22.16 (-10.62)	9.53 (-5.36)	2.64 (+0.79)	6.81 (+0.20)	25.38 (+4.94)	1.74 (+0.77)	2.22 (-0.34)	22.65 (+10.28)	5.7s (+2.1s)	[-0.5-0.5s] (-2s)

Supplementary Table 4: Tukey's multiple comparisons statistics results for Fig. 4g (Ctrl siRNA), post two-way ANOVA. P values were adjusted for multiple comparison using statistical hypothesis testing.

Tukey's multiple comparisons test	Mean Diff.	95.00% CI of diff.	Summary	Adjusted P Value
Nuc:[5-20s] vs. Nuc:(20-50s]	3.977	-1.423 to 9.378	ns	0.2599
Nuc:[5-20s] vs. Nuc:>50s	5.996	0.5954 to 11.40	*	0.0231
Nuc:[5-20s] vs. CCM:[5-20s]	-18.53	-23.93 to -13.13	****	<0.0001
Nuc:[5-20s] vs. CCM:(20-50s]	-1.218	-6.618 to 4.183	ns	0.9958
Nuc:[5-20s] vs. CCM:>50s	4.426	-0.9748 to 9.826	ns	0.1613
Nuc:[5-20s] vs. FTC:[5-20s]	-6.902	-12.30 to -1.501	**	0.0069
Nuc:[5-20s] vs. FTC:(20-50s]	-15.84	-21.24 to -10.44	****	<0.0001
Nuc:[5-20s] vs. FTC:>50s	-7.956	-13.36 to -2.556	**	0.0017
Nuc:(20-50s] vs. Nuc:>50s	2.019	-3.382 to 7.419	ns	0.9157
Nuc:(20-50s] vs. CCM:[5-20s]	-22.5	-27.90 to -17.10	****	<0.0001
Nuc:(20-50s] vs. CCM:(20-50s]	-5.195	-10.60 to 0.2056	ns	0.0647
Nuc:(20-50s] vs. CCM:>50s	0.4483	-4.952 to 5.849	ns	>0.9999
Nuc:(20-50s] vs. FTC:[5-20s]	-10.88	-16.28 to -5.479	****	<0.0001
Nuc:(20-50s] vs. FTC:(20-50s]	-19.82	-25.22 to -14.42	****	<0.0001
Nuc:(20-50s] vs. FTC:>50s	-11.93	-17.33 to -6.533	****	<0.0001
Nuc:>50s vs. CCM:[5-20s]	-24.52	-29.92 to -19.12	****	<0.0001
Nuc:>50s vs. CCM:(20-50s]	-7.213	-12.61 to -1.813	**	0.0045
Nuc:>50s vs. CCM:>50s	-1.57	-6.971 to 3.830	ns	0.9788

Nuc:>50s vs. FTC:[5-20s]	-12.9	-18.30 to -7.497	****	<0.0001
Nuc:>50s vs. FTC:(20-50s]	-21.84	-27.24 to -16.44	****	<0.0001
Nuc:>50s vs. FTC:>50s	-13.95	-19.35 to -8.552	****	<0.0001
CCM:[5-20s] vs. CCM:(20-50s]	17.31	11.91 to 22.71	****	<0.0001
CCM:[5-20s] vs. CCM:>50s	22.95	17.55 to 28.35	****	<0.0001
CCM:[5-20s] vs. FTC:[5-20s]	11.62	6.224 to 17.02	****	<0.0001
CCM:[5-20s] vs. FTC:(20-50s]	2.684	-2.717 to 8.084	ns	0.716
CCM:[5-20s] vs. FTC:>50s	10.57	5.169 to 15.97	****	<0.0001
CCM:(20-50s] vs. CCM:>50s	5.643	0.2427 to 11.04	*	0.0367
CCM:(20-50s] vs. FTC:[5-20s]	-5.684	-11.08 to -0.2837	*	0.0348
CCM:(20-50s] vs. FTC:(20-50s]	-14.62	-20.03 to -9.224	****	<0.0001
CCM:(20-50s] vs. FTC:>50s	-6.739	-12.14 to -1.338	**	0.0086
CCM:>50s vs. FTC:[5-20s]	-11.33	-16.73 to -5.927	****	<0.0001
CCM:>50s vs. FTC:(20-50s]	-20.27	-25.67 to -14.87	****	<0.0001
CCM:>50s vs. FTC:>50s	-12.38	-17.78 to -6.982	****	<0.0001
FTC:[5-20s] vs. FTC:(20-50s]	-8.941	-14.34 to -3.540	***	0.0005
FTC:[5-20s] vs. FTC:>50s	-1.055	-6.455 to 4.346	ns	0.9984
FTC:(20-50s] vs. FTC:>50s	7.886	2.485 to 13.29	**	0.0018

Supplementary Table 5: Tukey's multiple comparisons statistics results for Fig. 4g (CLCa siRNA) after two-way ANOVA. P values were adjusted for multiple comparison using statistical hypothesis testing.

Tukey's multiple comparisons test	Mean Diff.	95.00% CI of diff.	Summary	Adjusted P Value
Nuc:[5-20s] vs. Nuc:(20-50s]	3.55	-4.236 to 11.34	ns	0.7944
Nuc:[5-20s] vs. Nuc:>50s	5.584	-2.202 to 13.37	ns	0.2882
Nuc:[5-20s] vs. CCM:[5-20s]	-16.08	-23.86 to -8.291	****	<0.0001
Nuc:[5-20s] vs. CCM:(20-50s]	-2.901	-10.69 to 4.885	ns	0.917
Nuc:[5-20s] vs. CCM:>50s	3.23	-4.556 to 11.02	ns	0.8621
Nuc:[5-20s] vs. FTC:[5-20s]	-5.417	-13.20 to 2.369	ns	0.3221
Nuc:[5-20s] vs. FTC:(20-50s]	-19.26	-27.05 to -11.48	****	<0.0001
Nuc:[5-20s] vs. FTC:>50s	-11.48	-19.27 to -3.695	**	0.0017
Nuc:(20-50s] vs. Nuc:>50s	2.034	-5.752 to 9.820	ns	0.989
Nuc:(20-50s] vs. CCM:[5-20s]	-19.63	-27.41 to -11.84	****	<0.0001
Nuc:(20-50s] vs. CCM:(20-50s]	-6.452	-14.24 to 1.334	ns	0.1526
Nuc:(20-50s] vs. CCM:>50s	-0.32	-8.106 to 7.466	ns	>0.9999
Nuc:(20-50s] vs. FTC:[5-20s]	-8.967	-16.75 to -1.181	*	0.0172
Nuc:(20-50s] vs. FTC:(20-50s]	-22.81	-30.60 to -15.03	****	<0.0001
Nuc:(20-50s] vs. FTC:>50s	-15.03	-22.82 to -7.245	****	<0.0001
Nuc:>50s vs. CCM:[5-20s]	-21.66	-29.45 to -13.88	****	<0.0001
Nuc:>50s vs. CCM:(20-50s]	-8.486	-16.27 to -0.6993	*	0.0267
Nuc:>50s vs. CCM:>50s	-2.354	-10.14 to 5.432	ns	0.9733

Nuc:>50s vs. FTC:[5-20s]	-11	-18.79 to -3.215	**	0.0026
Nuc:>50s vs. FTC:(20-50s]	-24.85	-32.63 to -17.06	****	<0.0001
Nuc:>50s vs. FTC:>50s	-17.07	-24.85 to -9.279	****	<0.0001
CCM:[5-20s] vs. CCM:(20-50s]	13.18	5.390 to 20.96	***	0.0004
CCM:[5-20s] vs. CCM:>50s	19.31	11.52 to 27.09	****	<0.0001
CCM:[5-20s] vs. FTC:[5-20s]	10.66	2.874 to 18.45	**	0.0036
CCM:[5-20s] vs. FTC:(20-50s]	-3.185	-10.97 to 4.601	ns	0.8706
CCM:[5-20s] vs. FTC:>50s	4.596	-3.190 to 12.38	ns	0.5208
CCM:(20-50s] vs. CCM:>50s	6.132	-1.654 to 13.92	ns	0.1949
CCM:(20-50s] vs. FTC:[5-20s]	-2.516	-10.30 to 5.271	ns	0.961
CCM:(20-50s] vs. FTC:(20-50s]	-16.36	-24.15 to -8.575	****	<0.0001
CCM:(20-50s] vs. FTC:>50s	-8.58	-16.37 to -0.7936	*	0.0246
CCM:>50s vs. FTC:[5-20s]	-8.647	-16.43 to -0.8613	*	0.0231
CCM:>50s vs. FTC:(20-50s]	-22.49	-30.28 to -14.71	****	<0.0001
CCM:>50s vs. FTC:>50s	-14.71	-22.50 to -6.925	****	<0.0001
FTC:[5-20s] vs. FTC:(20-50s]	-13.85	-21.63 to -6.059	***	0.0002
FTC:[5-20s] vs. FTC:>50s	-6.064	-13.85 to 1.722	ns	0.205
FTC:(20-50s] vs. FTC:>50s	7.781	-0.004815 to 15.57	ns	0.0502

Supplementary Table 6: Tukey's multiple comparisons statistics results of differences between Ctrl and CLCa targeting siRNA from Fig. 4g, post two-way ANOVA. P values were adjusted for multiple comparison using statistical hypothesis testing.

Tukey's multiple comparisons test	Mean Diff.	95.00% CI of diff.	Summary	Adjusted P Value
**Nuc**				
CTRL [5-20s] vs. CTRL (20-50s]	3.977	-1.776 to 9.730	ns	0.3202
CTRL [5-20s] vs. CTRL >50s	5.996	0.2426 to 11.75	*	0.0369
CTRL [5-20s] vs. CLCa [5-20s]	0.7478	-5.005 to 6.501	ns	0.9987
CTRL [5-20s] vs. CLCa (20-50s]	4.298	-1.455 to 10.05	ns	0.242
CTRL [5-20s] vs. CLCa >50s	6.332	0.5787 to 12.09	*	0.0239
CTRL (20-50s] vs. CTRL >50s	2.019	-3.735 to 7.772	ns	0.8954
CTRL (20-50s] vs. CLCa [5-20s]	-3.229	-8.983 to 2.524	ns	0.5477
CTRL (20-50s] vs. CLCa (20-50s]	0.3209	-5.432 to 6.074	ns	>0.9999
CTRL (20-50s] vs. CLCa >50s	2.355	-3.399 to 8.108	ns	0.8187
CTRL >50s vs. CLCa [5-20s]	-5.248	-11.00 to 0.5052	ns	0.0909
CTRL >50s vs. CLCa (20-50s]	-1.698	-7.451 to 4.056	ns	0.947
CTRL >50s vs. CLCa >50s	0.3361	-5.417 to 6.089	ns	>0.9999
CLCa [5-20s] vs. CLCa (20-50s]	3.55	-2.203 to 9.304	ns	0.4442
CLCa [5-20s] vs. CLCa >50s	5.584	-0.1691 to 11.34	ns	0.0614
CLCa (20-50s] vs. CLCa >50s	2.034	-3.719 to 7.787	ns	0.8924
**CCM**				
CTRL [5-20s] vs. CTRL (20-50s]	17.31	11.56 to 23.06	****	<0.0001
CTRL [5-20s] vs. CTRL >50s	22.95	17.20 to 28.70	****	<0.0001

CTRL [5-20s] vs. CLCa [5-20s]	3.196	-2.557 to 8.949	ns	0.5587
CTRL [5-20s] vs. CLCa (20-50s]	16.37	10.62 to 22.13	****	<0.0001
CTRL [5-20s] vs. CLCa >50s	22.5	16.75 to 28.26	****	<0.0001
CTRL (20-50s] vs. CTRL >50s	5.643	-0.1101 to 11.40	ns	0.0572
CTRL (20-50s] vs. CLCa [5-20s]	-14.11	-19.87 to -8.359	****	<0.0001
CTRL (20-50s] vs. CLCa (20-50s]	-0.9361	-6.689 to 4.817	ns	0.9963
CTRL (20-50s] vs. CLCa >50s	5.196	-0.5575 to 10.95	ns	0.0964
CTRL >50s vs. CLCa [5-20s]	-19.76	-25.51 to -14.00	****	<0.0001
CTRL >50s vs. CLCa (20-50s]	-6.579	-12.33 to -0.8260	*	0.0172
CTRL >50s vs. CLCa >50s	-0.4474	-6.201 to 5.306	ns	0.9999
CLCa [5-20s] vs. CLCa (20-50s]	13.18	7.423 to 18.93	****	<0.0001
CLCa [5-20s] vs. CLCa >50s	19.31	13.55 to 25.06	****	<0.0001
CLCa (20-50s] vs. CLCa >50s	6.132	0.3786 to 11.89	*	0.031
**FTC**				
CTRL [5-20s] vs. CTRL (20-50s]	-8.941	-14.69 to -3.187	***	0.0005
CTRL [5-20s] vs. CTRL >50s	-1.055	-6.808 to 4.699	ns	0.9935
CTRL [5-20s] vs. CLCa [5-20s]	2.232	-3.521 to 7.986	ns	0.8491
CTRL [5-20s] vs. CLCa (20-50s]	-11.61	-17.37 to -5.860	****	<0.0001
CTRL [5-20s] vs. CLCa >50s	-3.832	-9.585 to 1.921	ns	0.3602
CTRL (20-50s] vs. CTRL >50s	7.886	2.133 to 13.64	**	0.0027
CTRL (20-50s] vs. CLCa [5-20s]	11.17	5.420 to 16.93	****	<0.0001

CTRL (20-50s] vs. CLCa (20-50s]	-2.673	-8.426 to 3.081	ns	0.7281
CTRL (20-50s] vs. CLCa >50s	5.109	-0.6444 to 10.86	ns	0.1063
CTRL >50s vs. CLCa [5-20s]	3.287	-2.466 to 9.040	ns	0.5288
CTRL >50s vs. CLCa (20-50s]	-10.56	-16.31 to -4.805	****	<0.0001
CTRL >50s vs. CLCa >50s	-2.777	-8.530 to 2.976	ns	0.6956
CLCa [5-20s] vs. CLCa (20-50s]	-13.85	-19.60 to -8.092	****	<0.0001
CLCa [5-20s] vs. CLCa >50s	-6.064	-11.82 to -0.3110	*	0.0338
CLCa (20-50s] vs. CLCa >50s	7.781	2.028 to 13.53	**	0.0031

Supplementary Table 7: Tukey's multiple comparisons statistics results for Fig. 5j, post two-way ANOVA. P values were adjusted for multiple comparison using statistical hypothesis testing.

Tukey's multiple comparisons test	Mean Diff.	95.00% CI of diff.	Summary	Adjusted P Value
Nuc: [5-20s] vs. Nuc:(20-50s]	5.726	-5.313 to 16.77	ns	0.6715
Nuc: [5-20s] vs. Nuc:>50s	7.492	-3.547 to 18.53	ns	0.3509
Nuc: [5-20s] vs. CCM: [5-20s]	-17.68	-28.72 to -6.645	***	0.0007
Nuc: [5-20s] vs. CCM:(20-50s]	0.9831	-10.06 to 12.02	ns	>0.9999
Nuc: [5-20s] vs. CCM:>50s	4.939	-6.100 to 15.98	ns	0.8094
Nuc: [5-20s] vs. FTC: [5-20s]	-5.714	-16.75 to 5.325	ns	0.6738
Nuc: [5-20s] vs. FTC:(20-50s]	-5.582	-16.62 to 5.457	ns	0.6984
Nuc: [5-20s] vs. FTC:>50s	-3.026	-14.06 to 8.013	ns	0.9851
Nuc:(20-50s] vs. Nuc:>50s	1.765	-9.274 to 12.80	ns	0.9996
Nuc:(20-50s] vs. CCM: [5-20s]	-23.41	-34.45 to -12.37	****	<0.0001
Nuc:(20-50s] vs. CCM:(20-50s]	-4.743	-15.78 to 6.296	ns	0.8392
Nuc:(20-50s] vs. CCM:>50s	-0.7869	-11.83 to 10.25	ns	>0.9999
Nuc:(20-50s] vs. FTC: [5-20s]	-11.44	-22.48 to -0.4010	*	0.0389
Nuc:(20-50s] vs. FTC:(20-50s]	-11.31	-22.35 to -0.2690	*	0.0423
Nuc:(20-50s] vs. FTC:>50s	-8.752	-19.79 to 2.287	ns	0.189
Nuc:>50s vs. CCM: [5-20s]	-25.18	-36.21 to -14.14	****	<0.0001
Nuc:>50s vs. CCM:(20-50s]	-6.509	-17.55 to 4.530	ns	0.5224
Nuc:>50s vs. CCM:>50s	-2.552	-13.59 to 8.487	ns	0.995

Nuc:>50s vs. FTC: [5-20s]	-13.21	-24.24 to -2.166	*	0.0125
Nuc:>50s vs. FTC:(20-50s]	-13.07	-24.11 to -2.034	*	0.0136
Nuc:>50s vs. FTC:>50s	-10.52	-21.56 to 0.5214	ns	0.0688
CCM: [5-20s] vs. CCM:(20-50s]	18.67	7.628 to 29.71	***	0.0004
CCM: [5-20s] vs. CCM:>50s	22.62	11.58 to 33.66	****	<0.0001
CCM: [5-20s] vs. FTC: [5-20s]	11.97	0.9311 to 23.01	*	0.0278
CCM: [5-20s] vs. FTC:(20-50s]	12.1	1.063 to 23.14	*	0.0256
CCM: [5-20s] vs. FTC:>50s	14.66	3.619 to 25.70	**	0.0048
CCM:(20-50s] vs. CCM:>50s	3.956	-7.083 to 15.00	ns	0.9319
CCM:(20-50s] vs. FTC: [5-20s]	-6.697	-17.74 to 4.342	ns	0.4873
CCM:(20-50s] vs. FTC:(20-50s]	-6.565	-17.60 to 4.474	ns	0.5118
CCM:(20-50s] vs. FTC:>50s	-4.009	-15.05 to 7.030	ns	0.9271
CCM:>50s vs. FTC: [5-20s]	-10.65	-21.69 to 0.3859	ns	0.0634
CCM:>50s vs. FTC:(20-50s]	-10.52	-21.56 to 0.5179	ns	0.0687
CCM:>50s vs. FTC:>50s	-7.965	-19.00 to 3.074	ns	0.2816
FTC: [5-20s] vs. FTC:(20-50s]	0.132	-10.91 to 11.17	ns	>0.9999
FTC: [5-20s] vs. FTC:>50s	2.688	-8.351 to 13.73	ns	0.993
FTC:(20-50s] vs. FTC:>50s	2.556	-8.483 to 13.59	ns	0.995

Supplementary Table 8: Key parameters and executable codes used for automated data processing.

<table>
<tr><th colspan="2">Parameters used in automatic processing</th></tr>
<tr><th colspan="2">CMEanalysis for frame rate(FR) 0.3s</th></tr>
<tr><td colspan="2">Example code run for control group:</td></tr>
<tr><td colspan="2">>> data = loadConditionData('/data/user/tnawara/Data/Data analysis/AA_First paper/Revisions/siRNA/Exp3/CLCa_CTRL_exp3', 'Ch1', 'Ch2', 'Ch3'}, 'EGFP', 'iRFP713', 'dZ'}, 'Parameters', [1.49 60 6.45e-6]);
>>[resCTRL, dataCTRL] = cmeAnalysis(data, 'ControlData', resCTRL, 'Overwrite', false, 'TrackingRadius', [1 3], 'TrackingGapLength', 13);</td></tr>
<tr><td colspan="2">Example code run for experimental group:</td></tr>
<tr><td colspan="2">>> data = loadConditionData('/data/user/tnawara/Data/Data analysis/AA_First paper/Revisions/siRNA/Exp3/CLCa_siRNA_exp3', 'Ch1', 'Ch2', 'Ch3'}, 'EGFP', 'iRFP713', 'dZ'}, 'Parameters', [1.49 60 6.45e-6]);
>>[resKD, dataKD] = cmeAnalysis(data, 'ControlData', resCTRL, 'Overwrite', false, 'TrackingRadius', [1 3], 'TrackingGapLength', 13);</td></tr>
<tr><td>1) 'Parameters'</td><td>[1.49 60 6.45e-6] / [NA Obj_Mag Camera_pix_size]</td></tr>
<tr><td>2) 'TrackingRadius'</td><td>[1 3]</td></tr>
<tr><td>3) 'TrackingGapLength'</td><td>13 (13*FR = 3.9s)
filtered and valid tracks Median Gap length = 1</td></tr>
<tr><td>4) Start and end track buffer @ runTrackProcessing.m</td><td>[15 15] (15*0.3 = 4.5s)</td></tr>
<tr><td>5) Minimum track lifetime @ runTrackProcessing.m</td><td>6*0.3 = 1.8s</td></tr>
<tr><th colspan="2">cme_wraper.m @ dz_beginning.m</th></tr>
<tr><td>1) Track length</td><td>≥ 5s</td></tr>
<tr><td>2) Single track with valid gaps</td><td>Category 1a (determined by CMEanalysis)</td></tr>
<tr><td>3) Is track iRFP713 and Δz positive</td><td>[1,1] (determined by CMEanalysis @ ProcessedTracks.mat -> tracks.significantSlave)</td></tr>
<tr><td>4) Numbers of the consecutive positive frame over the background to count signal beginning</td><td>5</td></tr>
<tr><td>5) Signal smoothing range for movmean</td><td>3</td></tr>
<tr><td>6) Frames below the threshold to count signal as de novo</td><td>3</td></tr>
<tr><td>7) Determining whether the signal is higher than background</td><td>signal + background > 2*SD + background</td></tr>
<tr><td>8) Quality of iRFP713 signal</td><td>above the threshold for more than 70% of EGFP signal</td></tr>
</table>

**cohort_wraper.m @ CCV_vs_FCL_graph_generator.m**	
1) Track length	≥ 5s
2) Single track with valid gaps	Category 1a (determined by CMEanalysis)
3) Is track iRFP713 and Δz positive	[1,1] (determined by CMEanalysis @ ProcessedTracks.mat -> tracks.significantSlave)
4) numbers of the consecutive positive frames over background to count signal beginning	5
5) Signal smoothing range for movmean	3
6) Frames below the threshold to count signal as de novo	3
7) Last n frames have to be under threshold	3
8) Amount of Δz frames above the threshold to count events as Δz positive	more than 30% of EGFP signal
9) Amount of Δz frames below the threshold to count events as Δz negative	100%
10) Mean and SD of Δz negative signal	(-25nm ≤ signal ≥25 nm)

Fig. 1e - Raw western blots: Green – CLCa, Red – GAPDH, <u>used in the manuscript</u>

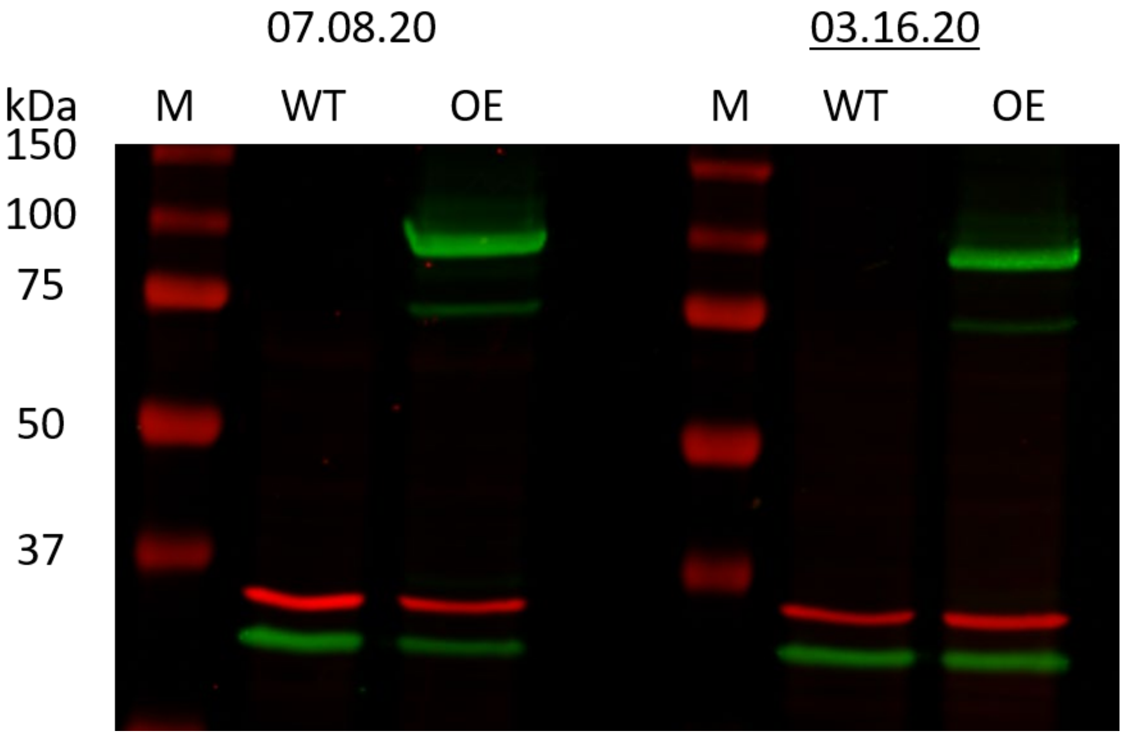

Fig. 4b - Raw western blots: Green – CLCa, Red – GAPDH, <u>used in the manuscript</u>

09.08.21

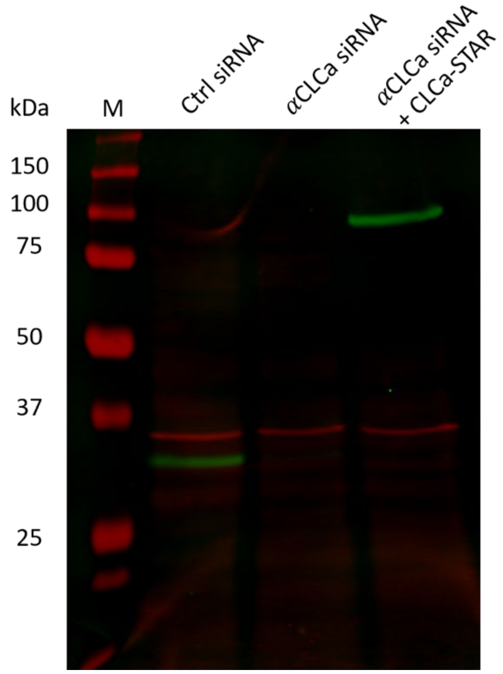

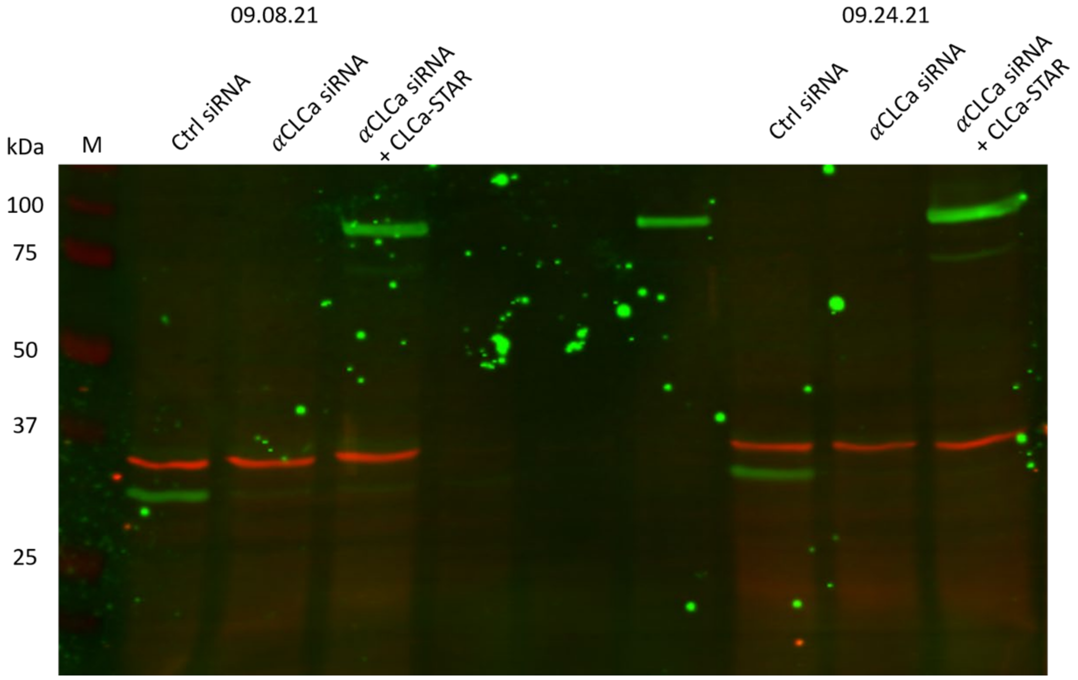

Fig. 5b - Raw western blots: Green – CLCa, Red – GAPDH, <u>used in the manuscript</u>

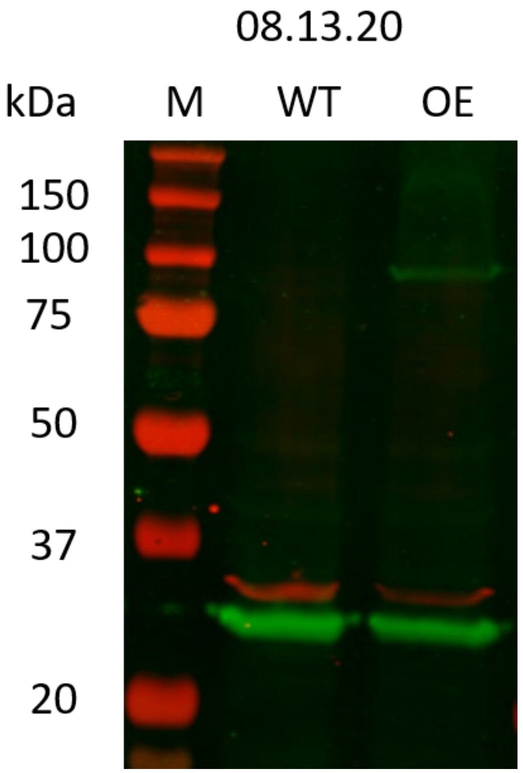

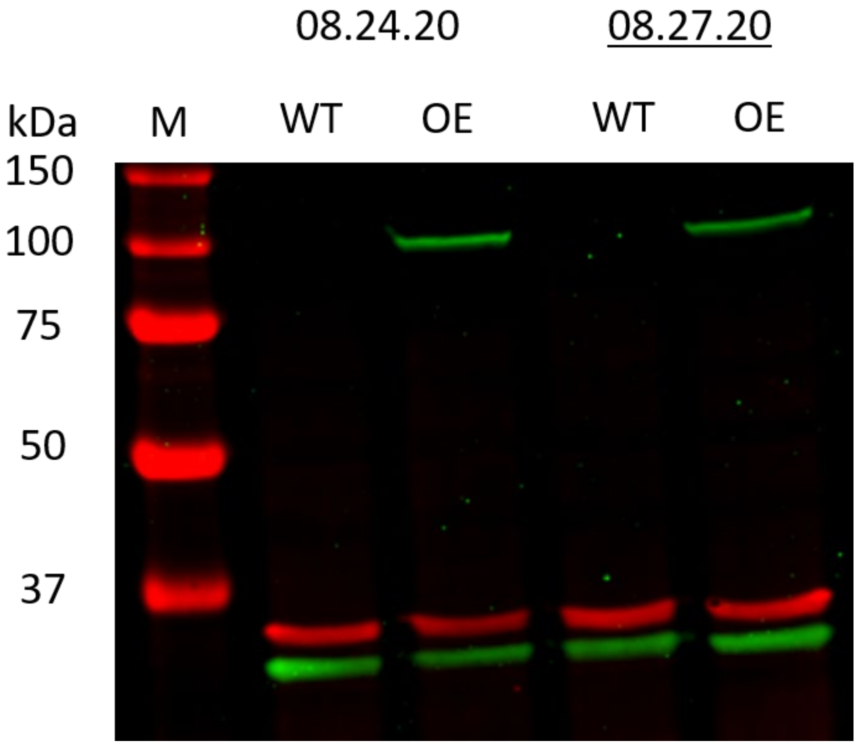

Supplement: Supplementary file 1 — Supplementary Information [file 41467_2022_29317_MOESM1_ESM.pdf]
