# Supplementary material for: Imaging vesicle formation dynamics supports the flexible model of clathrin-mediated endocytosis
Source: Nat Commun. 2022 Apr 1;13:1732. doi: 10.1038/s41467-022-29317-1 (PMC8976038; doi:10.1038/s41467-022-29317-1)
Supplement: Supplementary file 3 — Description of Additional Supplementary Files [file 41467_2022_29317_MOESM3_ESM.docx]

Description of Additional Supplementary Files

File name: Supplementary Movie 1

Description: STAR microscopy - Live-cell imaging of Cos-7 cell expressing CLCaiRFP713-EGFP (TIRF488 [cyan] & TIRF647 [magenta] overlay) and curvature (fire), scale bar = 20 μm and 5 μm for the inset. Small squares indicate events classified as curved, small dotted squares indicate events classified as flat by cohort_wrapper.m

File name: Supplementary Movie 2

Description: EPI/STAR microscopy - Live-cell imaging of Cos-7 cell expressing CLCa-iRFP713-EGFP with STAR (TIRF488 [cyan], TIRF647 [magenta]), epi (EPI488 [grays]), and curvature (fire), scale bar = 20 μm and 5 μm for the inset.

File name: Supplementary Movie 3

Description: Clathrin coat bending model classification - Live-cell imaging of Cos-7 cell expressing CLCaiRFP713-EGFP (TIRF488 [cyan] & TIRF647 [magenta] overlay) and curvature (fire), scale bar = 20 μm and 5 μm for the inset. Full squares indicate events classified as CCM, dotted squares indicate events classified as FTC, small dotted squares indicate events classified as Nuc by cme_wrapper.m
