# Supplementary material for: Imaging vesicle formation dynamics supports the flexible model of clathrin-mediated endocytosis
Source: Nat Commun. 2022 Apr 1;13:1732. doi: 10.1038/s41467-022-29317-1 (PMC8976038; doi:10.1038/s41467-022-29317-1)
Supplement: Supplementary file 7 — Reporting Summary [file 41467_2022_29317_MOESM7_ESM.pdf]

nature research

Corresponding author(s): Alexa Mattheyses, PhD

Last updated by author(s): Jan 26, 2022

Reporting Summary

Nature Research wishes to improve the reproducibility of the work that we publish. This form provides structure for consistency and transparency in reporting. For further information on Nature Research policies, see our Editorial Policies and the Editorial Policy Checklist.

Statistics

For all statistical analyses, confirm that the following items are present in the figure legend, table legend, main text, or Methods section.

n/a	Confirmed	
☐	☒	The exact sample size (n) for each experimental group/condition, given as a discrete number and unit of measurement
☐	☒	A statement on whether measurements were taken from distinct samples or whether the same sample was measured repeatedly
☐	☒	The statistical test(s) used AND whether they are one- or two-sided<br>*Only common tests should be described solely by name; describe more complex techniques in the Methods section.*
☒	☐	A description of all covariates tested
☐	☒	A description of any assumptions or corrections, such as tests of normality and adjustment for multiple comparisons
☐	☒	A full description of the statistical parameters including central tendency (e.g. means) or other basic estimates (e.g. regression coefficient) AND variation (e.g. standard deviation) or associated estimates of uncertainty (e.g. confidence intervals)
☐	☒	For null hypothesis testing, the test statistic (e.g. F, t, r) with confidence intervals, effect sizes, degrees of freedom and P value noted<br>*Give P values as exact values whenever suitable.*
☒	☐	For Bayesian analysis, information on the choice of priors and Markov chain Monte Carlo settings
☒	☐	For hierarchical and complex designs, identification of the appropriate level for tests and full reporting of outcomes
☒	☐	Estimates of effect sizes (e.g. Cohen's d, Pearson's r), indicating how they were calculated

Our web collection on statistics for biologists contains articles on many of the points above.

Software and code

Policy information about availability of computer code

Data collection	Nikon Elements (version 5.02); Coherent Connection (version 3.0.0.8); Image Studio (version 4.0)
Data analysis	FiJi (version 2.1.0/1.53c); Cairn Image Splitter plugin (version 1.5 - https://imagej.nih.gov/ij/plugins/cairn-splitter.html); MATLAB (version 2018b); MATLAB (version 2020b); optimized CMEanalysis software, custom MATLAB codes and custom FiJi Δz generator plugin deposited on lab GitHub (https://github.com/Mattheyses-Lab/Nawara_et_al._NatCommun_2022.git)

For manuscripts utilizing custom algorithms or software that are central to the research but not yet described in published literature, software must be made available to editors and reviewers. We strongly encourage code deposition in a community repository (e.g. GitHub). See the Nature Research guidelines for submitting code & software for further information.

Data

Policy information about availability of data

All manuscripts must include a data availability statement. This statement should provide the following information, where applicable:

- Accession codes, unique identifiers, or web links for publicly available datasets
- A list of figures that have associated raw data
- A description of any restrictions on data availability

Sample data sets are available on the lab GitHub (https://github.com/Mattheyses-Lab/Nawara_et_al._NatCommun_2022.git). All data supporting the findings of this study are available from the corresponding author on reasonable request. Source data are provided with this paper.

Field-specific reporting

Please select the one below that is the best fit for your research. If you are not sure, read the appropriate sections before making your selection.

☒ Life sciences ☐ Behavioural & social sciences ☐ Ecological, evolutionary & environmental sciences

For a reference copy of the document with all sections, see nature.com/documents/nr-reporting-summary-flat.pdf

Life sciences study design

All studies must disclose on these points even when the disclosure is negative.

Sample size	No statistical test or power analysis were used to predetermine sample size. The number of endocytic events (sample size) was proportional to those published previously (i.e. Loerke et al., (2009). doi.org/10.1371/journal.pbio.1000057). Moreover lifetime distribution (quality control) of endocytic events was in agreement with previously published data (Aguet et al., (2013). 10.1016/j.devcel.2013.06.019) indicating a sufficient sample size to accommodate for underlying data variation.
Data exclusions	Cells were excluded from analysis if the signal to noise of fluorescent intensity ratio (curvature/Δz channel) was low.
Replication	All experiments were replicated at least two-three times and successfully verified reproducibility of the outcomes.
Randomization	Randomization was not applicable to the study, as the study did not involve clinical trials, assignment to experimental groups, or subjective analysis. For cell data transfected cells with similar amount of expression were chosen for analysis, due to specifics of CMEanalysis detection.
Blinding	Blinding was not applicable to the study as data grouping and analysis were performed in an unbiased manner using CMEanalysis and custom written codes. Data was analyzed in agreement with common strategies.

Reporting for specific materials, systems and methods

We require information from authors about some types of materials, experimental systems and methods used in many studies. Here, indicate whether each material, system or method listed is relevant to your study. If you are not sure if a list item applies to your research, read the appropriate section before selecting a response.

Materials & experimental systems

n/a	Involved in the study	
☐	☒	Antibodies
☐	☒	Eukaryotic cell lines
☒	☐	Palaeontology and archaeology
☒	☐	Animals and other organisms
☒	☐	Human research participants
☒	☐	Clinical data
☒	☐	Dual use research of concern

Methods

n/a	Involved in the study	
☒	☐	ChIP-seq
☒	☐	Flow cytometry
☒	☐	MRI-based neuroimaging

Antibodies

Antibodies used	rabbit anti-CLTA (Proteintech, 10852-1-AP; 1:1000);<br>mouse anti-GAPDH (Cell Signaling Technology, 97166; 1:1000);<br>goat anti-mouse IgG (Li-Cor, 925-68020, IRDye 680LT; 1:20,000);<br>goat anti-rabbit IgG (Li-Cor, 925-32211, IRDye 800CW; 1:15,000).
Validation	All antibodies were validated by the manufacture:<br>rabbit anti-CLTA - https://www.ptglab.com/products/CLTA-Antibody-10852-1-AP.htm<br>mouse anti-GAPDH - https://www.cellsignal.com/products/primary-antibodies/gapdh-d4c6r-mouse-mab/97166<br>goat anti-mouse IgG - https://www.licor.com/bio/reagents/irdye-680lt-goat-anti-mouse-igg-secondary-antibody<br>goat anti-rabbit IgG - https://www.licor.com/bio/reagents/irdye-800cw-goat-anti-rabbit-igg-secondary-antibody

Eukaryotic cell lines

Policy information about cell lines

Cell line source(s)	Cos-7 cells (ATCC, CRL-1651); HUVECs (Pooled HUVECs, Lonza, C2519A)
Authentication	None of the cell lines used were authenticated.

Mycoplasma contamination	The cell lines tested negative for mycoplasma contamination.
Commonly misidentified lines<br>(See ICLAC register)	No commonly misidentified lines were used in this study.
